# Efficacy of Platelet-Rich Plasma in Post-extraction Healing of Mandibular Third Molars: A Clinical and Radiographic Evaluation

**DOI:** 10.7759/cureus.82891

**Published:** 2025-04-24

**Authors:** Navneet Singh, Rohit Goyal, Lakhan Talreja, Niyutna Sheshamani, Aditi Mugalikar, Sagar R Dixit, Rashi Kaushik

**Affiliations:** 1 Department of Oral and Maxillofacial Surgery, Maharaja Ganga Singh Dental College and Research Centre, Sri Ganganagar, IND; 2 Department of Oral and Maxillofacial Surgery, Derma Dental, Navi Mumbai, IND; 3 Department of Oral and Maxillofacial Surgery, Dr. G.D. Pol Foundation Yerala Medical Trust (YMT) Dental College and Hospital, Navi Mumbai, IND; 4 Department of Medicine, Government Medical College and Hospital, Sri Ganganagar, IND

**Keywords:** extraction, platelet rich plasma, sockets, third molars, wound healing

## Abstract

Introduction: Healing of extraction sockets is a critical factor in oral and maxillofacial surgery, particularly when preserving alveolar bone and periodontal stability is essential. Platelet-rich plasma (PRP), an autologous concentrate of growth factors, has gained attention for its potential to enhance bone regeneration and soft-tissue healing. This study aimed to evaluate the clinical and radiographic outcomes of PRP application in extraction sockets after impacted mandibular third molar surgery.

Materials and methods: Forty patients who underwent impacted mandibular third molar extraction were divided into two groups according to clinical decision-making in conjunction with patient preference: the PRP group, in which PRP was applied in the extraction socket, and the non-PRP group, which received standard post-extraction care. Clinical parameters, including probing depth and wound closure, and radiographic assessments of alveolar bone level and bone density were recorded at baseline, one month, three months, and six months postoperatively.

Results: The application of PRP resulted in significant improvements in alveolar bone preservation and periodontal health, with a notable reduction in probing depth compared to the non-PRP group. Although PRP did not show a significant difference in bone density between the groups, a sustained increase over time suggested a positive effect on bone remodeling. PRP also accelerated wound healing, with initial dehiscence observed, but improved closure by day seven, indicating a biphasic influence on tissue repair.

Conclusion: PRP demonstrated potential benefits in enhancing soft and hard tissue healing after mandibular third molar extraction. Its ability to promote periodontal stability and wound closure suggests its clinical utility for oral surgical procedures. However, larger randomized controlled trials with extended follow-up are needed to establish standardized protocols and confirm their long-term efficacy.

## Introduction

Surgical extraction of impacted third molars, commonly referred to as wisdom teeth, is one of the most frequently performed procedures in oral and maxillofacial surgery [1]. While this intervention is often necessary to address pathologies such as pericoronitis, caries, or crowding, it is not without complications. The most prevalent postoperative concerns are delayed wound healing, pain, alveolar osteitis (dry socket), and development of periodontal defects, particularly on the distal aspect of the adjacent second molars [2]. Regeneration of soft and hard tissues at the surgical site plays a crucial role in mitigating these complications and achieving optimal clinical outcomes.

In recent years, there has been growing interest in the application of autologous platelet-rich plasma (PRP) to enhance wound healing and bone regeneration following oral surgical procedures [3]. PRP is a biologically active autologous concentrate derived from the patient’s own blood and contains a platelet concentration several times higher than that found in the baseline plasma. It was prepared by centrifugation to isolate the platelet-rich buffy coat layer. This layer is rich in a variety of growth factors that are key mediators of the tissue repair and regeneration processes [4].

Growth factors released from the alpha granules of activated platelets, including platelet-derived growth factor (PDGF), transforming growth factor-beta (TGF-β), insulin-like growth factor-1 (IGF-1), and fibroblast growth factor (FGF), orchestrate complex cellular events such as chemotaxis, proliferation, differentiation, angiogenesis, and extracellular matrix synthesis [4,5]. These events are fundamental to the healing cascade of the soft and hard tissues. Specifically, PDGF stimulates the proliferation of mesenchymal cells, whereas TGF-β regulates bone matrix formation and remodeling [4].

The application of PRP has gained significant traction in various medical disciplines, including cardiac surgery, neurosurgery, and, more recently, oral and maxillofacial surgery [3,4]. In oral surgery, PRP has been used as an adjunct to bone grafts, in sinus lift procedures, and in the management of periapical lesions and osseous defects around dental implants [6]. Its benefits include accelerated soft tissue healing, enhanced bone regeneration, and reduced postoperative complications, such as infection and delayed healing.

The role of PRP in enhancing extraction socket healing remains controversial owing to inconsistent clinical findings across studies. Some studies have reported improved early soft tissue healing, reduced postoperative pain, and enhanced bone regeneration with PRP application in extraction sockets [7,8]. Conversely, studies by Gürbüzer et al. [9] and Arenaz-Búa et al. [10] found no statistically significant difference in bone density or healing outcomes when comparing PRP-treated sites to controls. These discrepancies are largely attributed to the variability in PRP preparation protocols, differences in platelet concentration, inconsistent use of activators such as thrombin or calcium chloride, and diverse clinical endpoints used to assess healing (clinical, radiographic, and histological parameters). Additionally, factors such as patient-specific variables (age, systemic health, and smoking) and limited sample sizes in some studies further complicate the ability to draw definitive conclusions. Hence, despite its promising biological potential, the clinical efficacy of PRP in extraction socket healing remains inconclusive, warranting further standardized, large-scale, randomized controlled trials.

Given the need for more definitive evidence, the present study was undertaken to assess the clinical and radiographic efficacy of autologous PRP gel in promoting bone regeneration and soft tissue healing following the surgical extraction of mandibular third molars over a six-month period. This study aimed to provide valuable insights into the role of PRP as a regenerative adjunct in routine oral surgery, with the ultimate goal of improving patient outcomes and minimizing postoperative complications.

## Materials and methods

Study design and setting

This prospective cohort study was conducted in the Department of Oral and Maxillofacial Surgery at the Maharaja Ganga Singh Dental College and Research Centre, Sri Ganganagar, Rajasthan. A total of 40 patients were enrolled from August 2023 to August 2024. The patients were allocated into two cohorts, PRP and non-PRP, based on clinical evaluation, operator preference, and patient consent rather than random assignment. As a cohort study, the focus was on observing outcomes following naturally occurring group allocation rather than controlled randomization. The study was approved by the Institutional Ethics Committee (MGSDC/SY/23/2) and followed the principles of the Declaration of Helsinki. Written informed consent was obtained from all patients before starting the study.

Patient eligibility

Healthy individuals between the ages of 18 and 50 years, of either sex, who required surgical removal of the impacted mandibular third molars, were considered for inclusion. Exclusion criteria included the presence of systemic conditions contraindicating extractions, such as uncontrolled diabetes or bleeding disorders; pregnant or lactating females; those who were taking medications that could impair wound healing; and those with a history of head and neck radiotherapy. The types of mandibular third molar impactions were evaluated preoperatively using the Pederson Difficulty Index [11], which considers three variables: spatial relationship (angulation of the tooth), depth of impaction relative to the occlusal plane, and ramus relationship (space available vs. space required). Each parameter is scored, and the total score classifies the surgical difficulty as minimal (3-4), moderate (5-7), or difficult (8-10). For the purpose of this study, only cases with minimal difficulty (total score 3-4) were included to ensure surgical standardization across both cohorts. Most impactions were mesioangular or vertical and positioned at a superficial level, with adequate space in the mandibular ramus to allow relatively uncomplicated surgical access. This selection was intended to minimize variability in healing outcomes due to procedural complexity.

Sample size estimation

G*Power software (Heinrich-Heine-Universität Düsseldorf, Düsseldorf, Germany) was used for sample size estimation. A minimum of 40 total samples was estimated considering an effect size of 0.80 for the mean difference in bone density (101.05) with a pooled standard deviation of 126.02 percent between the PRP and non-PRP groups [12]. The power of the study was 80% with an alpha error of 5%.

Cohort allocation

Forty eligible patients were prospectively enrolled and assigned to one of two cohorts. The allocation was determined by clinical decision-making in conjunction with patient preference. No randomization process was used. Patients who agreed to the additional venous blood draw required for PRP preparation, and when PRP preparation resources were available, were included in the PRP cohort. Conversely, patients who declined blood draw or when PRP facilities were unavailable were assigned to the non-PRP cohort. This allocation method ensured that the treatment choices mirrored those in everyday clinical settings. Patients in the PRP cohort (n = 20) received PRP gel application to the extraction socket following third molar removal, whereas patients in the non-PRP cohort (n = 20) underwent standard surgical extraction without PRP gel placement.

PRP gel preparation

**Figure 1:**
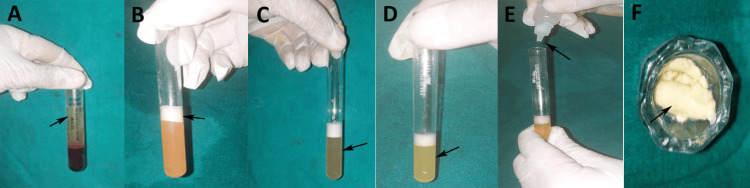
Platelet-rich plasma (PRP) gel preparation. A: whole venous blood at first centrifugation at 1000 rpm for 10 min with formation of supernatant containing PRP; B: PRP, buffy coat, and the upper 1–2 mm layer of red blood cells transferred into a fresh tube; C: at second centrifugation at 1000 rpm for 10 min; D: the upper layer was discarded and the remaining lower layer mixed thoroughly to obtain concentrated PRP (cPRP); E: cPRP added with 10% calcium chloride for activation; F: formation of PRP gel. This figure illustrates the preparation of PRP gel from venous blood drawn from a patient in the PRP cohort (arrows marking the stages), with the patient’s informed consent for its use.

For PRP gel preparation, 8 mL of venous blood was drawn from patients in the PRP cohort under aseptic conditions and transferred to vacutainers containing acid citrate dextrose (ACD) as an anticoagulant. Blood samples were processed using a REMI R-8C Laboratory Centrifuge (REMI Elektrotechnik Ltd., Mumbai, India). The first centrifugation was performed at 1000 rpm for 10 min to separate the plasma, buffy coat, and blood cells (Figure 1A). The plasma and buffy coat layer, along with the upper 1-2 mm layer of red blood cells, were transferred to a fresh sterile vacutainer (Figure 1B) and subjected to a second centrifugation at 1000 rpm for another 10 min (Figure 1C). The upper layer of the supernatant was discarded, and the remaining concentrate was thoroughly mixed to yield approximately 4 mL of concentrated PRP (cPRP) (Figure 1D). Activation of PRP was achieved by mixing 2 mL of cPRP with 0.06 mL of 10% calcium chloride (CaCl₂), using either a sterile syringe or a dropper (Figure 1E). The remaining 2 mL of cPRP was added to this activated solution, and the mixture was incubated in a dry bath incubator (Eurocon Instruments Pvt. Ltd., Chennai, Tamil Nadu) at 750°C for 15 min to form a PRP gel (Figure 1F).

Surgical procedure

**Figure 2:**
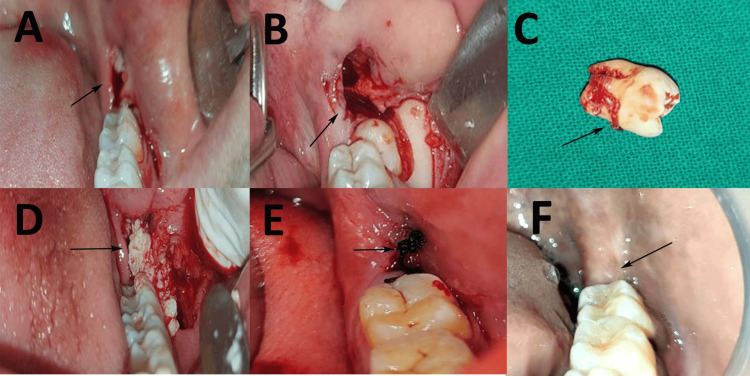
Surgical removal of mandibular third molar with platelet-rich plasma (PRP) gel placement in the PRP cohort. A: crestal incision; B: crestal with sulcular incision to raise the flap; C: removal of mandibular left third molar; D: placement of PRP gel; E: flap closure with sutures; F: postoperative healing. This figure illustrates surgical removal with the placement of PRP gel into the extraction socket of a patient in the PRP cohort (arrows marking the stages), with the patient’s informed consent for its use.

All procedures were performed under local anesthesia using 2% lignocaine with 1:80,000 adrenaline. A full-thickness mucoperiosteal flap was raised to access the impacted mandibular third molar, with the incision extending from the retromolar area to the distal surface of the second molar (Figure 2A), followed by a sulcular and release incision (Figure 2B). Bone removal and tooth sectioning were performed using a Lindemann carbide bur and a diamond bur (EdgeEndo, New Mexico, USA) under continuous saline irrigation. After extraction (Figure 2C), PRP gel was placed into the sockets of patients in the PRP cohort (Figure 2D), whereas no PRP was applied in the non-PRP cohort. The surgical sites were sutured using 3-0 silk sutures (Figure 2E), which were removed on the seventh postoperative day (Figure 2F). Postoperative care, including antibiotics (amoxicillin with clavulanic acid), analgesics (ibuprofen 800 mg daily for five days), and 0.12% chlorhexidine mouthwash, was standardized across both cohorts.

Outcome assessment

Clinical and radiographic parameters were assessed both preoperatively and during follow-up over six months at standardized intervals (one week, one month, three months, and six months). The probing depth distal to the second molar was measured using a UNC-15 periodontal probe (Hu-Friedy, Chicago, Illinois, USA) with mm markings, ensuring that the probe was placed parallel to the long axis of the second molar to maintain consistency. Each measurement was recorded to the nearest mm by the same calibrated examiner to minimize inter-examiner variability. Wound dehiscence was evaluated by visual inspection and classified as present or absent using a predefined clinical checklist during the postoperative follow-up.

**Figure 3:**
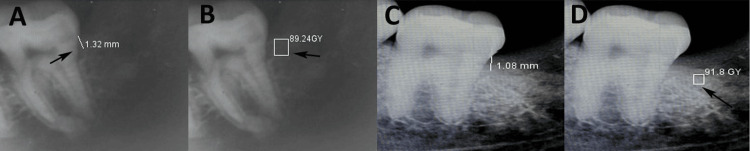
Assessment of alveolar bone level (ABL) and bone density (BD) in grayscale values in platelet-rich plasma (PRP) cohort A: ABL at three months; B: BD at three months; C: ABL at six months; D: BD at six months. This figure illustrates the intraoral periapical radiographic assessment of ABL and BD of a patient in the PRP cohort, with the patient’s informed consent for its use.

For radiographic evaluation, standardized intraoral periapical radiographs were obtained using a long-cone paralleling technique with customized bite blocks to ensure the reproducibility of the radiographs at each follow-up. The alveolar bone level (ABL) was assessed from the cementoenamel junction (CEJ) of the second molar to the alveolar crest distal to the second molar using digital calipers with radiographic software (ImageJ, NIH, Bethesda, MD, USA) (Figures 3A, 3C). Bone density was measured using grayscale analysis within the same software, standardizing contrast and brightness settings across all images to avoid discrepancies (Figures 3B, 3D).

To enhance the reliability of radiographic measurements, intra-examiner reliability testing was performed. The examiner re-measured 20% of randomly selected radiographs after a two-week interval, and intra-class correlation coefficients (ICCs) were calculated, with values above 0.80 considered acceptable for measurement consistency.

Statistical analysis

Descriptive and analytical statistics were computed using the Stata Statistical Software Release 18 (StataCorp LLC, College Station, TX, USA). The normality of the data was checked using the Shapiro-Wilk test, and the data was found to be normally distributed. The chi-square test evaluated wound healing dehiscence between groups, while repeated analysis of variance (ANOVA) compared bone density, ABL, and probing depth in the socket across time points. A post-hoc analysis was performed using the Tukey test. Intergroup comparisons were performed using independent t-tests. Significance was set at p < 0.05.

## Results

The results showed high intra-examiner reliability (r) with an ICC value of 0.83. Both groups consisted of eight (40%) males and 12 (60%) females with a mean age of 29.54 ± 4.36 years. The comparison of bone density between the groups at different time intervals showed no statistically significant differences preoperatively (p = 0.731), at three months postoperatively (p = 0.773), or at six months postoperatively (p = 0.803). However, within-group analysis using repeated-measures ANOVA indicated a significant increase in bone density over time in both groups (p = 0.001). These results indicated that while the intervention did not lead to a significant difference between the groups, both groups experienced significant bone density improvements over time. This suggests a potential positive effect of natural healing or treatment interventions within each group, warranting further investigation of long-term outcomes (Table 1).

**Table 1:** Intragroup and intergroup comparison of bone density (grayscale values) for different time intervals. *p-value < 0.05: significant, PRP: platelet-rich plasma gel; ANOVA: analysis of variance. Data is presented in the form of mean and standard deviation (SD).

Grayscale value	Cohort without PRP	Cohort with PRP	t value	p-value using an independent t-test
	Mean ± SD	Mean ± SD		
Preoperative	80.67 ± 15.68	78.95 ± 15.76	0.35	0.731
Postoperative at 3-month	81.54 ± 15.63	80.11 ± 15.48	0.29	0.773
Postoperative at 6-month	82.52 ± 15.53	81.30 ± 15.21	0.25	0.803
F value	8.21	55.89		
p-value using repeated ANOVA	0.001*	0.001*		

The post-hoc pairwise comparison of bone density within the groups showed significant differences across time points. In both groups, there was a statistically significant increase in bone density from the preoperative period to three months postoperatively (p < 0.001) and from the preoperative period to six months postoperatively. Additionally, the increase in bone density between three and six months postoperatively was significant in the cohort with PRP (p < 0.001) but not in the cohort without PRP (p = 0.244). These findings suggest that bone density progressively improved over time in both groups, with a more pronounced and sustained improvement in the PRP cohort. The lack of a significant difference between the three-month and six-month values in the cohort without PRP indicates a possible plateau in bone healing, whereas the cohort with PRP continued to show significant improvement, highlighting the potential effect of PRP (Table 2).

**Table 2:** Post-hoc pairwise comparison of bone density for study groups using the Tukey test. *p-value < 0.05: significant; PRP: platelet-rich plasma gel; MD: mean difference.

Pairwise groups		Cohort without PRP			Cohort with PRP		
		MD	t value	p-value	MD	t value	p-value
Preoperative	Postoperative at 3-month	-0.87	-6.25	0.001*	-1.16	-6.83	0.001*
Preoperative	Postoperative at 6-month	-1.85	-3.25	0.012*	-2.35	-8.18	0.001*
Postoperative at 3-month	Postoperative at 6-month	-0.98	-1.84	0.244	-1.19	-6.18	0.001*

Preoperatively, the ABL was significantly greater in the cohort with PRP than in the cohort without PRP (p = 0.007). At three months postoperatively, there was further improvement in ABL in both groups, with a significantly greater improvement in the cohort with PRP (p = 0.001). At six months postoperatively, ABL continued to improve significantly in both groups, with the cohort with PRP showing a more pronounced improvement (p = 0.001). Repeated-measures ANOVA showed a statistically significant change in ABL over time in both groups (p = 0.001). These findings suggest that alveolar bone remodeling occurred progressively postoperatively, with a greater degree of bone remodeling in the PRP cohort (Table 3).

**Table 3:** Intragroup and intergroup comparison of alveolar bone levels (ABL) (in mm) for different time intervals. *p-value < 0.05: significant, PRP: platelet-rich plasma gel; ANOVA: analysis of variance. Data is presented in the form of mean and standard deviation (SD).

ABL in mm	Cohort without PRP	Cohort with PRP	t value	p-value using an independent t-test
	Mean ± SD	Mean ± SD		
Preoperative	2.99 ± 0.40	2.59 ± 0.50	2.83	0.007*
Postoperative at 3-month	2.46 ± 0.41	1.84 ± 0.44	4.65	0.001*
Postoperative at 6-month	1.97 ± 0.35	1.15 ± 0.35	7.32	0.001*
F value	257.23	207.74		
p-value using repeated ANOVA	0.001*	0.001*		

The post-hoc pairwise comparison of ABL between different time intervals within both groups showed significant changes over time. In the cohort without PRP, there was a statistically significant improvement in ABL from preoperatively to three months postoperatively (p < 0.001) and from preoperatively to six months postoperatively (p < 0.001). Similarly, in the cohort with PRP, ABL improved significantly between the preoperative period and three months postoperatively (p < 0.001) and between the preoperative period and six months postoperatively (p < 0.001). The comparison between the three-month and six-month postoperative intervals also showed a significant improvement in ABL for both groups (p < 0.001). These results indicated a progressive improvement in ABL over time, with a more pronounced improvement in the PRP cohort (Table 4).

**Table 4:** Post-hoc pairwise comparison of alveolar bone levels (ABL) for study groups using the Tukey test. *p-value < 0.05: significant; PRP: platelet-rich plasma gel; MD: mean difference.

Pairwise groups		Cohort without PRP			Cohort with PRP		
		MD	t value	p-value	MD	t value	p-value
Preoperative	Postoperative at 3-month	0.53	13.54	0.001*	0.75	14.60	0.001*
Preoperative	Postoperative at 6-month	1.02	18.90	0.001*	1.44	15.48	0.001*
Postoperative at 3-month	Postoperative at 6-month	0.50	12.16	0.001*	0.69	11.37	0.001*

The comparison of probing depths at different sites (distal, distobuccal, and distolingual) between the groups revealed significant reductions postoperatively. At the distal site, there was no significant difference between the groups preoperatively (P = 0.747). However, at three and six months postoperatively, the probing depth significantly decreased in the cohort with PRP compared to that in the cohort without PRP (p = 0.001). Similarly, at the distobuccal site, the preoperative probing depth did not differ significantly between the groups (p = 0.215), but at three and six months postoperatively, there was a significant reduction in the cohort with PRP. At the distolingual site, the preoperative probing depth was comparable between the groups (p = 0.887), whereas a significant reduction was observed at three and six months postoperatively in the PRP cohort. Repeated ANOVA confirmed significant intragroup reductions in probing depth over time across all sites (p = 0.001). These results indicate that surgical intervention led to a significant reduction in probing depth over time, with greater improvement in the PRP cohort. These findings suggested enhanced periodontal healing and reduced pocket depth, emphasizing the effectiveness of PRP in improving periodontal health (Table 5).

**Table 5:** Intragroup and intergroup comparison of probing depth (PD) (in mm) for different time intervals. *p-value < 0.05: significant; PRP: platelet-rich plasma gel; ANOVA: analysis of variance. Data is presented in the form of mean and standard deviation (SD).

PD at various time intervals	PD at distal			PD at distobuccal			PD at distolingual		
	Cohort without PRP	Cohort with PRP	p-value using an independent t-test	Cohort without PRP	Cohort with PRP	p-value using an independent t-test	Cohort without PRP	Cohort with PRP	p-value using an independent t-test
	Mean ± SD	Mean ± SD		Mean ± SD	Mean ± SD		Mean ± SD	Mean ± SD	
Preoperative	4.95 ± 0.94	5.05 ± 1.00	0.747	6.25 ± 0.97	6.65 ± 1.04	0.215	4.90 ± 0.97	4.85 ± 1.23	0.887
Postoperative at 3-month	4.70 ± 0.98	3.00 ± 1.03	0.001*	5.50 ± 0.76	4.50 ± 1.19	0.003*	4.45 ± 0.89	2.70 ± 0.92	0.001*
Postoperative at 6-month	3.35 ± 1.18	1.25 ± 0.44	0.001*	3.60 ± 0.82	1.50 ± 0.76	0.001*	2.75 ± 0.91	1.05 ± 0.22	0.001*
F value	30.66	196.85		101.5	249.67		67.09	132.01	
p-value using repeated ANOVA	0.001*	0.001*		0.001*	0.001*		0.001*	0.001*	

On day one, there was no significant difference in dehiscence between groups (p = 0.667). However, by day two, a significantly higher proportion of the cohort with PRP exhibited wound dehiscence than the cohort without PRP (p = 0.011). By day seven, the trend reversed, with a significantly higher number of cohorts without PRP showing wound dehiscence, whereas most cohorts with PRP had healed (p = 0.001). These results indicate that while initial healing may be delayed in the cohort treated with PRP, long-term wound closure is significantly better, suggesting improved healing outcomes with PRP (Table 6).

**Table 6:** Association of dehiscence of wound with the study groups using the chi-square test. *p-value < 0.05: significant; PRP: platelet-rich plasma gel Data is presented in the form of n (%).

Dehiscence	Postoperative day 1		Postoperative day 2		Postoperative day 7	
	Cohort without PRP	Cohort with PRP	Cohort without PRP	Cohort with PRP	Cohort without PRP	Cohort with PRP
Present	17 (85%)	18 (90%)	6 (30%)	14 (70%)	19 (95%)	6 (30%)
Absent	3 (15%)	2 (10%)	14 (70%)	6 (30%)	1 (5%)	14 (70%)
Chi stat	0.23		6.4		18.03	
p-value	0.667		0.011*		0.001*	

## Discussion

The present study investigated the effect of PRP on bone density, ABL, probing depth, and wound healing following surgical intervention. The results indicated that, while PRP did not lead to statistically significant differences in bone density between the groups, it did contribute to a more sustained increase in bone density over time. Furthermore, PRP was associated with a significantly greater improvement in ABL and probing depth as well as improved wound healing outcomes over the six-month postoperative period.

Our findings align with previous studies indicating that PRP promotes bone regeneration by releasing growth factors such as PDGF, TGF-β, and vascular endothelial growth factor (VEGF), which facilitate osteogenesis and angiogenesis [3,9,10,12]. PRP is the autologous concentration of platelets within a diminutive volume of plasma. It is classified as a first-generation concentrate that employs calcium glutamate/thrombin to induce coagulation and an anticoagulant solution comprising citrate phosphate dextrose adenine (CPDA) [13]. Following activation via thrombin or calcium chloride, platelets contained within PRP secrete a variety of crucial growth factors that have been documented to be synthesized by platelets [14]. These growth factors encompass three isomers of PDGF (PDGFαα, PDGFββ, and PDGFαβ), two distinct TGF-β isoforms (TGFβ1 and TGFβ2), as well as epithelial growth factor and VEGF [15]. When applied locally, platelet concentrates enhance the generation of osteoprogenitor cells, initiate osteoblast activity, expedite epithelialization of the gingiva, facilitate cellular recruitment to the surgical site, and promote angiogenesis.

Although natural healing processes can account for the observed increase in bone density in both groups, the sustained improvement in the PRP cohort suggests a prolonged effect on osteoblastic activity. This finding supports the hypothesis that PRP enhances the osteoconductive and osteoinductive properties of bone grafts or surgical interventions, although its effects may be more evident in long-term follow-ups [16]. Future studies with extended follow-up periods are warranted to determine if the observed benefits persist beyond six months.

The study found a significant improvement in ABL over time in both groups. At three and six months postoperatively, ABL was significantly greater in the PRP cohort than in the control cohort, indicating a more substantial preservation of the alveolar bone. These findings align with those of previous research highlighting PRP’s role in reducing bone resorption and enhancing bone remodeling by stimulating mesenchymal stem cell differentiation and promoting mineralization [10,12,17,18]. Moreover, the progressive improvement in ABL between three and six months in the PRP cohort suggests that PRP may help sustain bone volume over time, which is crucial for preventing postoperative complications [3,9].

The results showed significant reductions in probing depth at different sites (distal, distobuccal, and distolingual) postoperatively in both groups, with greater improvement in the PRP cohort. This finding suggests that PRP may accelerate periodontal healing by enhancing soft tissue regeneration and reducing inflammation, which aligns with previous studies [3,19]. The observed improvement in probing depth is consistent with previous studies reporting that PRP contributes to improved periodontal attachment and reduced probing depth, likely owing to its ability to enhance fibroblast proliferation and extracellular matrix synthesis [19,20]. Additionally, the sustained improvement in the PRP cohort may be attributed to PRP’s anti-inflammatory properties, which help maintain a favorable periodontal environment postoperatively [21].

This study found no significant difference in wound dehiscence between the groups on day one. However, by day two, a significantly higher proportion of the PRP cohort had wound dehiscence. Interestingly, by day seven, the trend reversed, with fewer PRP patients exhibiting dehiscence than the control group, indicating enhanced wound closure. The initial delay in healing in the PRP cohort may be attributed to the early inflammatory response stimulated by PRP, which can increase vascular permeability and transiently exacerbate tissue edema [22]. However, PRP promotes accelerated soft tissue healing through the sustained release of growth factors that enhance epithelialization, collagen deposition, and neovascularization [15].

These findings support the clinical utility of PRP in improving long-term wound healing outcomes, particularly in high-risk patients with compromised healing potential (such as those with diabetes, smokers, or those with osteoporosis) [23]. Future studies should explore the molecular mechanisms underlying PRP’s biphasic effect on wound healing to optimize its application in clinical practice.

## Conclusions

This study demonstrated that PRP enhanced alveolar bone remodeling, periodontal healing, and wound closure following the surgical extraction of impacted mandibular third molars. While PRP did not significantly alter bone density between the groups, its sustained improvement over time suggests a potential role in promoting long-term osteogenesis. The significant reduction in probing depth and superior alveolar bone preservation in the PRP cohort highlight its ability to enhance periodontal stability and minimize postoperative bone resorption. Additionally, PRP accelerated wound healing, with a higher initial incidence of dehiscence, followed by superior closure by day seven, indicating its biphasic effect on tissue repair. These findings support the clinical utility of PRP as an adjunct in oral and maxillofacial surgeries to improve surgical outcomes, particularly in high-risk patients.
